# 

**Published:** 1877-01

**Authors:** 


### BOOKS AND PAMPHLETS RECEIVED.

A Treatise on Hernia; with a new process for its Radical Cure, etc., etc. By Greensville Dowell, M.D., etc. 1876.

Yellow Fever and Malarial Diseases, embracing a History of the Epidemics of Yellow Fever in Texas, etc., etc. By Greensville Dowell, M.D., etc.

Modern Therapeutics; A Compendium of Recent Formulæ, Approved Treatment and Specific Methods in Medicine and Surgery. By Geo. H. Napheys, A.M., MD., etc. New edition. 1877.

Contributions to Reparative Surgery. By Gurdon Buck, M.D., etc.

On Coughs, Consumption, and Diet in Disease. By Horace Dobell, M.D., etc. Edited by D. G. Brinton, M.D. 1877.

Clinical Studies illustrated by Cases observed in Hospital and

Private Practice. By Sir John Rose Cormack, K.B., etc. Two volumes. 1876.

Liver Complaint, Nervous Dyspepsia and Headache, etc. By M. L. Holbrook, M.D., etc. 1876.

Lectures on the Physical Diagnosis of Diseases of the Heart. By Arthur Ernest Sansom, M.D., etc. 1876.

Ophthalmic and Otic Memoranda. By D. B. St. J. Roosa, M.D., etc., and E. F. Ely, M.D., etc. 1876.

Walsh's Physicians' Combined Call-Book and Tablet. Second edition.

A Report on the Death-rate of Each Sex in Michigan, etc. By H. B. Baker, M.D., etc.

Prescription and Clinic Record, with Blanks for Prescriptions. Prepared by E. Seguin, M.D.

Annual Report of the Surgeon General U. S. A. 1876.

International Uniformity in the Practice and Records of Physic. By E. Seguin, M.D.

Transactions of the Colorado State Medical Society. Sixth Annual Convention. 1876.

### ANNOUNCEMENTS FOR THE MONTH.

**MONDAYS.** SOCIETIES.
*Mondays, Jan.* 1 *and* 5.—Chicago Medical Society. Regular meetings.
*Mondays, Jan.* 8 *and* 22.—Chicago Soc. of Phys. and Surgeons. Regular meetings.
CLINICS. *Every Monday.*
At Eye and Ear Infirmary, 2 P. M.—Prof. Holmes.
At Central Dispensary (Wood and Harrison sts.)—2 P.M. *Gynecological*, Dr. Adolphus; 3 P.M. *Diseases of Children*, Dr. R. S. Hall.
At Mercy Hospital—2 P. M., *Surgical*, Prof. Andrews.
At Rush College—2¼ P. M., *Medical*, Dr. Bridge.
At Chicago College, 2 P. M *Gynecological*—Prof. Merriman.
LECTURES. *Every Monday.*
At Rush Medical College (Harrison and Wood sts.)—9½ to 12½ o'clock, Profs. Freer, Lyman and Powell; 4 to 6, Profs. Gunn and Haines. At Chicago College—8½ to 12½, Profs. Jewell, Hyde, Hatfield and Bond; 3 to 6, Profs. Nelson and Davis, Quine and Andrews, and Roler. At Woman's College—8½ to 11½, Profs. Hotz, Marguerat and Earle; 3 to 5, Profs. Paoli and Stevenson.

**TUESDAYS.** SOCIETIES.
*Tuesday, Jan.* 9.—Academy of Science. Regular meeting at 8 P.M. (263 Wabash av.)
CLINICS. *Every Tuesday.*
At Eye and Ear Infirmary—2 P. M., Prof. Jones.
At County Hospital—2 P. M., *Medical*, Prof. Ross. 3 P. M., *Surgical*, Prof. Bogue.
At Mercy Hospital—2 P. M., *Medical*, Prof. Hollister.
At Chicago College—2 P. M., *Gynecological*, Prof. Roler.
At Central Dispensary—2, *Surgical*, Dr. Graham.
LECTURES. *Every Tuesday.*
At Rush College—9½ to 12½, Profs. Miller, Allen and Parkes; 4 to 6, Profs. Gunn and Haines. At Chicago College—8½ to 12½, Profs. Jewell, Quine, Merriman and Bond; 3 to 6, Profs. Johnson and Nelson, Andrews and Hatfield, and Byford. At Woman's College—9 to 12, Profs. Bartlett, Curtis and Fitch; 3 and 5, Profs. Hayes and Macdonald.

**WEDNESDAYS.** CLINICS. *Every Wednesday.*
At Eye and Ear Infirmary—2 P.M., Prof. Holmes.
At County Hospital—2 P. M., *Gynecological*, Prof. Fitch; 2 P. M., *Ophthalmological*, Dr. Montgomery.
At Chicago College—2 P. M., *Gynecological*, Prof. Nelson.
At Mercy Hospital—2 P. M., *Ophthalmological*, Prof. Jones.
At Central Dispensary—3 P. M., *Dermatological*, Dr. Maynard.
LECTURES. *Every Wednesday.*
At Rush College—9½ to 12½, Profs. Freer, Allen and Parkes; 4 to 6, Profs. Etheridge and Haines. At Chicago College—8½ to 12½, Profs. Hyde, Isham, Hatfield and Bond; 3 to 6, Profs. Davis and Curtis, Quine and Jones, and Roler. At Woman's College—8:30 to 12, Profs. Marguerat, Hotz and Blake; 3 to 6, Paoli, Graham and Stevenson.

**THURSDAYS.** CLINICS. *Every Thursday.*
At Eye and Ear Infirmary—2 P. M., Prof. Hotz.
At Mercy Hospital—2 P. M., *Medical*, Prof. Davis.
At Rush College—2 P. M., *Medical*, Prof. Ross; 3 P. M., *Diseases of the Nervous System*, Prof. Lyman.
At Chicago College—2 P. M., *Gynecological*, Prof. Merriman.
At Central Dispensary—2 P.M., *Surgical*, Dr. Graham.
LECTURES. *Every Thursday.*
At Rush College—9½ to 12½, Profs. Miller, Allen and Parkes; 4 to 6, Profs. Gunn and Etheridge. At Chicago College—8½ to 12½, Profs. Hollister, Isham, Merriman and Bond; 3 to 6, Profs. Johnson and Nelson, Andrews and Hatfield, and Byford. At Woman's College—9 to 12, Profs. Bartlett, Curtis and Fitch; 4 to 6, Profs. Macdonald and Thompson.

**FRIDAYS.** SOCIETIES.
*Friday, Jan.* 12.—State Microscopical Society of Illinois. Regular meeting, 8 P. M.
CLINICS. *Every Friday.*
At County Hospital—2 P. M., *Medical*, Prof. Ross; 3 P. M., *Surgical*, Prof. Bogue.
At Mercy Hospital—2 P. M., *Medical*, Prof. Davis.
At Chicago College—2 P. M., *Gynecological*, Prof. Roler.
At Central Dispensary—2 P. M., *Gynecological*, Dr. Adolphus; 3 P.M., *Diseases of Children*, Dr. R. S. Hall.
LECTURES. *Every Friday.*
At Rush College—9½ to 12½, Profs. Freer, Allen and Parkes; 4 to 6, Profs. Gunn and Holmes. At Chicago College—8½ to 12½, Profs. Hollister, Isham, Hatfield and Bond; 3 to 6. Profs. Davis and Curtis, Jones and Quine, and Roler. At Woman's College—9 to 12, Profs. Dyas, Marguerat and Earle; 3 to 5, Prof. Hayes; 5, Prof. Stevenson.

**SATURDAYS.** CLINICS. *Every Saturday.*
At Chicago College—2 P. M., *Surgical*, Prof. Andrews or Isham; *Gynecological*, Prof. Nelson; 3 P. M., *Medical*, Prof. Johnson.
At Rush College—2 P. M., *Surgical*, Prof. Gunn.
LECTURES. *Every Saturday.*
At Rush College—8½ to 12½, Profs. Lyman, Miller, Etheridge and Parks. At Chicago College, 8½ to 11½, Profs. Hollister, Quine and Hyde; 3 to 6, Profs. Nelson and Johnson, Andrews and Quine and Byford. At Woman's College—9 to 11, Profs. Bartlett, Earle and Fitch; 3 and 5, Profs. Paoli, Thompson and Macdonald.

At all the above named Clinics visiting regular practitioners are, we believe, admitted.
At the South Side Dispensary (Chicago College) there are six daily special Clinics, for sections of the classes of the Chicago College.

